# Awareness of and Challenges in Utilizing the Ayushman Bharat Digital Mission for Healthcare Delivery: Qualitative Insights from University Students in Coastal Karnataka in India

**DOI:** 10.3390/healthcare13040382

**Published:** 2025-02-11

**Authors:** Rajesh Kamath, Muneera Banu, Nagaraj Shet, Varshini R. Jayapriya, Vani Lakshmi Ramesh, Selim Jahangir, Nahima Akthar, Helmut Brand, Vidya Prabhu, Vishwajeet Singh, Sagarika Kamath

**Affiliations:** 1 Department of Healthcare and Hospital Management, Prasanna School of Public Health, Manipal Academy of Higher Education, Manipal 576104, India; rajesh.kamath@manipal.edu (R.K.); muneera.psphmpl2022@learner.manipal.edu (M.B.); varshini.psphmpl2023@learner.manipal.edu (V.R.J.); 2 Department of International Health, Care and Public Health Research Institute—CAPHRI, Faculty of Health, Medicine and Life Sciences, Maastricht University, 6211 LK Maastricht, The Netherlands; helmut.brand@maastrichtuniversity.nl (H.B.); dr.sagarikarkamath@gmail.com (S.K.); 3 Department of Hospital Administration, Yenepoya Medical College Hospital, Yenepoya University, Mangaluru 575018, India; 4 Department of Health Technology and Informatics, Prasanna School of Public Health, Manipal Academy of Higher Education, Manipal 576104, India; vani.lakshmi@manipal.edu; 5 Transdisciplinary Centre for Qualitative Methods & Department of Social and Health Innovation, Prasanna School of Public Health, Manipal Academy of Higher Education, Manipal 576104, India; 6 Directorate of Online Education, Manipal Academy of Higher Education, Manipal 576104, India; nahima.a@manipal.edu (N.A.); vishwajeet.singh@manipal.edu (V.S.); 7 Department of Global Public Health Policy and Governance, Prasanna School of Public Health, Manipal Academy of Higher Education, Manipal 576104, India; vidya.p@manipal.edu

**Keywords:** Ayushman Bharat Digital Mission, digital health, healthcare challenges, digital literacy, digital healthcare integration

## Abstract

**Background:** The Ayushman Bharat Digital Mission (ABDM) aims to enhance healthcare delivery in India through digital integration. The ABDM, an essential part of India’s healthcare system, aspires to transform healthcare delivery through digitization, by emphasizing affordability, accessibility, and transparency. This qualitative study investigated the awareness, challenges, and perceptions of the ABDM among postgraduate students in coastal Karnataka, focusing on their experiences and interactions with digital health technologies. **Methodology:** A qualitative descriptive approach was employed, involving semi-structured interviews with 17 purposively selected participants from a health science university. The interviews were audio-recorded, transcribed, and analyzed using the NVivo 12 software for thematic analysis. **Results:** This study revealed that, while many students recognized the ABDM’s goal of establishing comprehensive digital health infrastructure to seamlessly integrate healthcare services and information systems, detailed knowledge about its functionalities, implementation processes, and challenges was limited. Participants highlighted the need for improved digital literacy and support to maximize the effective use of the ABDM. **Conclusions:** The successful implementation of the ABDM depends on overcoming major challenges such as poor awareness and concerns about data security. Addressing these issues will require focused educational campaigns, increased accessibility, and joint efforts by the government, healthcare providers, and technological partners.

## 1. Introduction

The broad idea of “digital health” includes the application of technology to enhance healthcare and well-being. These technologies range from simple wearables and portable mobile phones to sophisticated satellite systems. The emergence of technologies associated with digital health has unlocked a multitude of possibilities to influence the direction of universal health coverage (UHC) and to guarantee the impact of public health policies and initiatives [1]. Gaining knowledge about the world’s finest practices in digital health will be of great help to better understand how e-health efforts have been carried out successfully throughout the world [2]. In the international context, Australia pioneered the adoption of a population-focused approach to implement My Health Record (MHR) in the years 2016–2017; this is a comprehensive national repository of patients’ electronic health records, aimed at simplifying the patient’s healthcare journey. This strategy offers valuable insights for India, where obtaining consent, preserving the privacy of health data, and safeguarding personal data are major concerns [3,4]. The National Health System (NHS) in the United Kingdom initiated its digital transformation journey in the month of July 2019 by introducing NHS electronic care records, enabling the secure exchange of patient information with their consent. This innovation simplified the workflow for general practitioners and other primary care staff. It facilitated appointments with hospital doctors’ electronic prescription transmission, the management of recurring medications, the registration of organ donors and the assessment of their symptoms, the exchange of patient data, and electronic referrals to specialized healthcare centers [5]. South Korea’s Ministry of Health, which has stringent data protection policies, implemented a master plan spanning two decades for the integration of information and communications technology (ICT) into their national health system. The successful integration of ICT solutions in South Korean healthcare facilities offers crucial insights for India, which is currently in the process of creating an advanced digital health claims platform [6].

The majority of healthcare providers and patients prefer traditional in-person/offline methods of delivering care, but physical lockdowns and restrictions during the COVID-19 pandemic raised the need for digital health solutions, highlighting the necessity of more effectively integrating digital technologies towards healthcare services in areas like remote clinical management, surveillance, and telemedicine [7,8]. In collaboration with the private sector, the National Informatics Centre, under the Ministry of Electronics and Information Technology, initiated the development of Aarogya Setu, a digital application that played a crucial role in steering the Indian government’s efforts to effectively address and manage the challenges created by the COVID-19 pandemic [9]. Communication and trust-building challenges, as well as infrastructure and policy concerns, all have an impact on the adoption and efficacy of digital health technologies. Overcoming these constraints is vital in facilitating equitable access, high-quality care, and the involvement of patients in digital health interventions [10]. The Digital Personal Data Protection Act (DPDP Act) was enacted in 2023, impacting pivotal components of India’s healthcare ecosystem, such as the ABDM and Ayushman Bharat–Pradhan Mantri Jan Aarogya Yojana (AB-PMJAY). The Act emphasizes resolving the significant challenges of ensuring data portability and interoperability among different consent managers [11]. India, with 1.2 billion mobile users and over 600 million smartphones, represents a unique opportunity to improve digital health [12]. By investing in 5G technology and upgrading the communication infrastructure in developing nations like India, there exists significant potential to enhance the landscape of digital health services and delivery [13].

India’s public health policies and initiatives are rapidly incorporating digital healthcare provision. The Indian government initiated the Ayushman Bharat Digital Mission (ABDM) in 2021 to establish a national digital health ecosystem. The government introduced the flagship program with a five-year, 1600-crore budget, spanning from 2021–2022 to 2025–2026. This mission focuses on building the necessary framework for the country’s integrated digital health infrastructure. It aims to digitally bridge the gap between various stakeholders in the healthcare ecosystem. In addition to aiding the closing of the digital gap, government welfare programs like the ABDM also make it easier for individuals to obtain healthcare in a timely, safe, economical, and accessible manner. It offers an aided and offline method to set up an Ayushman Bharat Health Account (ABHA) in places with inadequate hardware or internet access. The ABHA app works as a digital archive for a person’s medical records. Users of the app can also communicate with registered healthcare experts. Under the ABDM, almost 235,000medical facilities have been validated in the Health Facility Registry. Of these healthcare facilities, more than 166,000 are government facilities, while 69,633 are private. Additionally, more than 284,000 healthcare professionals are involved in the program. The ABDM, which was introduced with the goal of giving Indians access to digital health services, is in line with programs like tele-mental health. It is a continuation of the NDHM and contributes to India’s efforts to digitalize healthcare. The National Digital Health Mission (NDHM), established by the Indian government, aims to improve health systems via the use of digital health technology. The four main areas in which the ABDM measures healthcare delivery are quality, cost, accessibility, and transparency. The Government of India has introduced the ABDM with the purpose of fostering healthcare digitalization and establishing an open, interoperable digital health ecosystem for the nation. Nevertheless, there are further issues to be considered, such as confidentiality, the acceptability of virtual help, accessibility for people with limited resources, unreliable internet or online platforms, and qualified health professionals [14,15,16]

The healthcare delivery system in India is adapting to align with the rapid advancements of the digitalization era [16]. In FY 2023-2024, the government allocated INR 341.02 crore to the ABDM to support its implementation and development [17]. Since 2016, India’s healthcare sector has experienced significant expansion, with a compound annual growth rate (CAGR) of approximately 22%. The hospital industry has shown substantial growth, increasing from USD 61.79 billion in FY 2017 to USD 132.84 billion in FY 2022, at a CAGR of 16–17%. The country’s medical technology market is expected to grow dramatically, reaching USD 50 billion by 2025. In 2019, the telemedicine market in India was worth approximately USD 830 million. It is expected to reach USD 5.5 billion by 2025, growing at a CAGR of 30.2% during 2022–2027. This robust growth and upward trend indicate the transformative potential of technological advancements and digital health innovations in enhancing healthcare accessibility, efficiency, and affordability in India’s healthcare ecosystem, while addressing persistent challenges in service delivery [18,19,20,21,22,23,24].

The global digital health market, which was valued at USD 255.87 billion in 2022, is expected to grow significantly, reaching USD 565.23 billion by 2030. This sector is expected to grow at a compound annual growth rate (CAGR) of 15.5% between 2023 and 2030 [25]. In the Indian context, the digital health market was reported to be valued at about USD 12.2 billion in 2023 and is projected to reach USD 25.64 billion by 2027, with a CAGR of 20.4% [26].

This growth, coupled with the government’s decision to allow 100% foreign direct investment through the automatic route, is making India a global destination for digital health investment [18,27].

In FY 2023-2024, the government allocated INR 341.02 crore to the ABDM for its implementation and development, which is a 70% increase compared to the previous year’s INR 200 crore [28].

Despite the increasing body of research on digital health technologies and their potential to improve healthcare delivery, there are still a number of unanswered questions about how Indian users perceive these healthcare initiatives. Although existing studies from countries like Australia, the UK, and South Korea provide valuable insights into effective digital health models, they overlook the sociocultural, infrastructural, and digital literacy challenges that are specific to India. Meanwhile, the previous studies in India have primarily focused on technical and policy-level frameworks, highlighting accessibility and affordability. However, the issues of infrastructure deficiencies, data privacy issues, and trust building regarding the ABDM are under-researched. In addition, studies on the awareness and attitudes of end users—especially postgraduate students in health-related fields, who will be the future implementers and supporters of digital health projects—are limited. Out of a total of 265,620,913 ABHAs, the majority are within the age range of 19 to 45 years [29]. Focusing on postgraduate students in hospital administration, this study offers nuanced insights into their awareness, perceptions, and challenges surrounding the ABDM. Targeting students in the 20–30 age group has a dual impact: not only do they become aware of the ABDM and its benefits, but they also act as conduits to spread this knowledge to their families, particularly their parents. This age group is highly familiar with smartphones and digital tools, making them effective adopters and promoters of digital healthcare initiatives. Moreover, incorporating health education, including information about the ABDM and related technologies, into the academic curriculum can ensure equitable access to essential healthcare knowledge.

In this context, this study will address the awareness, perceptions, and challenges among postgraduate students in coastal Karnataka regarding the Ayushman Bharat Digital Mission (ABDM). Thus, this study aims to explore the perspectives and experiences of university students regarding the ABDM, with an emphasis on uncovering knowledge gaps and barriers to adoption.

## 2. Methodology

### Study Design

This study adopted a qualitative descriptive approach to explore and understand the perspectives and experiences of postgraduate students in a private university in coastal Karnataka, India, regarding the ABDM. This approach was chosen for its ability to provide insights into the participants. The utilization of in-depth interviews, supported by tools such as the NVivo 12 software for thematic analysis, allowed for a nuanced exploration of the participants’ experiences, thereby contributing valuable insights to the discourse surrounding the implementation and the impact of the ABDM.

## 3. Data Collection Procedures

This study utilized a semi-structured in-depth interview guide to collect data. A total of 17 postgraduate students pursuing a Master’s in Hospital Administration (MHA) participated. The participants were recruited using purposive sampling to ensure diversity in terms of educational backgrounds. Interviews were conducted in person at mutually agreed-upon places. For instance, some of the participants were interviewed in the library premises, a few in hostels, and some in playgrounds. The interviews were conducted between December 2023 and January 2024. On average, the interviews lasted between 40 and 50 min. Participants provided informed consent, confirming their voluntary participation and understanding of the study’s goals and procedures.

An interview guide was developed based on the research questions and research objectives and was validated through a pilot study. Open-ended questions were used to elicit participants’ experiences and perspectives related to digital health innovations (see Supplementary Materials). All interviews were audio-recorded and later transcribed using Microsoft Teams. The collected data were stored securely, and confidentiality was maintained throughout the process.

## 4. Data Analysis

The transcribed interview data were analyzed thematically using the NVivo 12 software. The thematic analysis approach was employed to identify key patterns, themes, and insights related to the participants’ experiences with the ABDM. The analysis involved the initial coding of the data, followed by code groups and the identification of broader themes that captured the essence of the participants’ responses. This process allowed for a nuanced exploration of the participants’ perspectives on the implementation and impact of the ABDM.

## 5. Ethical Considerations

The Institutional Ethics Committee approved this study on 21 November 2023 (IEC2-604/2023). Ethical approval was obtained from the relevant authorities at the study site. All participants were informed of the study’s purpose, the data collection methods, and their rights. Confidentiality was maintained by anonymizing all participant data, and participants were informed that they could withdraw from the study at any time without consequence.

## 6. Trustworthiness of the Study

To ensure the trustworthiness of the research, criteria such as credibility and transferability were addressed. The interview guide was pilot-tested to ensure the clarity and relevance of the questions. Transferability was maintained by providing rich, detailed descriptions of the study context and participant experiences.

## 7. Results

This study included 17 participants, ranging in age from 23 to 32, belonging to various geographic locations and educational levels (Table 1). The majority of the participants were in their mid-20s to early 30s, and there were slightly more women (10) than men (7). The majority of the participants were from Karnataka (7), Kerala (5), Tamil Nadu (3), and Maharashtra (2), indicating the predominance of regional locations in southern and coastal India. Academically, the participants were from a variety of healthcare-related and non-healthcare fields, and most of them had medical and allied health degrees, including a Bachelor of Medicine and Surgery (MBBS), a Bachelor of Homeopathic Medicine and Surgery (BHMS), a Bachelor of Naturopathy and Yogic Sciences (BNYS), a Bachelor of Dental Surgery (5 participants), a Bachelor of Physical Therapy or Physiotherapy (2), and Cardiac Perfusion Technology.

The findings revealed a significant gap in the participants’ understanding of government initiatives; although they expressed considerable interest in staying informed about technological advancements in healthcare, the participants demonstrated a widespread lack of awareness regarding government programs aimed at transforming the nation’s healthcare system. Key challenges identified included limited exposure, knowledge barriers, communication gaps, information complexity, and a lack of perceived importance. Most participants misunderstood the ABDM for the AB-PMJAY health insurance scheme. This misinterpretation highlights the need for clearer communication and more effective promotion surrounding government programs and activities. It implies that the ABDM’s identity and mission are not well defined in the public’s perception, resulting in misinformation and limiting its potential impact.

The analysis resulted in six major themes: (1) ABDM overview and awareness, (2) advantages and benefits of the ABDM, (3) public awareness and education gaps, (4) data security and privacy, (5) user adoption and usage, and (6) drawbacks and concerns. Table 2 gives an overview of the major themes and sub-themes regarding the awareness of and barriers in utilizing digital healthcare facilities under the ABDM.

### 7.1. Theme 1: ABDM Overview and Awareness

There was a general lack of understanding regarding the ABDM among the study participants; this is one of the factors contributing to the inadequate utilization of the ABDM. Many individuals mistake it for the AB-PMJAY insurance program and are not aware of its primary goal, which is to digitize medical records. Some individuals only come across information about the ABDM through indirect channels, such as through notifications from government authorities or because of their involvement in healthcare services. This reflects a knowledge gap regarding the distinct purpose of the ABDM, which focuses on digitizing healthcare records and creating a unified digital health ecosystem.

“*Uh, I first got to know about Ayushman digital mission, because my father runs a hospital. It’s a pediatric setup. So, after second COVID-19 wave, there was a circulation from the district collector in my district, Sagar district in Madhya Pradesh. So, in that it was clearly mentioned that all the healthcare institutes, healthcare providers, even the hospital administrators have to register themselves*.” (P9, 28, Male)

The participants emphasized that there has been inadequate promotion and communication about the ABDM compared to the extensive awareness campaigns conducted for the AB-PMJAY. The lack of visible advertisements and outreach initiatives appears to have contributed to poor public recognition. Participant 9’s awareness stemmed from a professional context, where specific instructions were communicated to healthcare institutions and professionals. This suggests that the awareness of the ABDM is limited to individuals with direct involvement in healthcare operations or institutions that receive government directives. The participants consistently indicated a need for greater efforts in promoting the ABDM, both to the public and within professional healthcare circles. Public campaigns like those for the AB-PMJAY could help to clarify the ABDM’s purpose and encourage adoption.

### 7.2. Theme 2: Advantages and Benefits of ABDM

The participants identified various advantages of the Ayushman Bharat Digital Mission (ABDM), emphasizing its potential to improve healthcare accessibility, streamline medical record management, and enhance patient outcomes. The ABDM’s teleconsultation services and remote diagnostic capabilities are seen as particularly beneficial for individuals in remote areas, the elderly, and those with mobility challenges, reducing the need for physical travel. The participants also noted that the digitalization of medical records alleviates the burden of managing extensive paperwork, especially for chronic patients requiring long-term care. The portability of digital health records was another significant benefit identified, as it allows patients to access healthcare services seamlessly across locations and during emergencies, ensuring continuity of care regardless of geographical constraints.

“*Our country will benefit from ABDM, like one is paperless records, and the medical records will be available everywhere. If they’re travelling somewhere else. If suddenly in case of any medical or emergency, they can visit any hospital, and they will be ready with their data. And they don’t even bother they are not familiar with medical terms, and they cannot describe exactly what condition they have. It will be easy for many people across India*.” (P11, 29, Male)

### 7.3. Theme 3: Public Awareness and Education Gaps

The participants emphasized the importance of enhancing the public’s awareness and addressing education gaps regarding the ABDM using widely accessible platforms and targeting specific demographics. A recurring suggestion was the use of social media as a key medium to disseminate information. The participants noted that most individuals are active on social media platforms, making it an effective tool to create awareness and improve the recognition of the ABDM. The widespread use of these platforms can ensure that information reaches a diverse audience and engages people regularly.

In addition to social media campaigns, educational institutions were identified as vital channels for awareness raising. A participant suggested that schools and colleges could play a critical role in bridging knowledge gaps by integrating awareness campaigns into their activities.

“*Targeting the age group of 16 to 24 through such initiatives can significantly enhance accessibility and awareness of health-related matters. Integrating health education about technologies like ABDM into the curriculum ensures that all individuals have equal access to essential healthcare information*.” (P17, 23, Female)

### 7.4. Theme 4: Data Security and Privacy

The results reveal a spectrum of attitudes towards sharing personal health information and adopting digital healthcare solutions. The participants expressed mixed views on the theme of data security and privacy in the context of the ABDM, highlighting both concerns and potential benefits. Several participants raised apprehensions about sharing sensitive health information. Many participants acknowledged that, even with advanced security measures, no system can guarantee 100% data protection, creating hesitation in fully trusting the platform. Many participants expressed a willingness to engage with the ABDM, drawing parallels with their experiences during the COVID-19 pandemic. They acknowledged that sharing personal health information is not a new concept, referencing prior experiences with the CoWIN platform during the COVID-19 pandemic, where they willingly shared personal information for public health purposes. Their familiarity with digital platforms for access to vaccination certificates suggests potential comfort with sharing health data if it contributes to improved healthcare access and outcomes.

“*So, if this is Ayushmann Bharat Digital Mission then like our complete health record will be. Digitalized, I think so. If it will be useful, then I’ll be comfortable sharing it*.” (P10, 26, Female)

Significant concerns were raised about the sensitivity of certain health information. One participant emphasized the reluctance to share mental health records, reflecting the stigma and fear associated with these disclosures.

“*I will not share, some aspects of health is very sensitive in nature and especially, I mean mental health is a very scary thing*.” (P2, 32, Female)

Another participant highlighted the potential misuse of sensitive health data, such as information about abortions or miscarriages, underscoring the vulnerability of individuals if data breaches occur. A recurring sentiment was that, even with advanced security measures, data cannot be guaranteed as 100% secure, creating apprehension about fully embracing the platform.

“*Health records sharing, no I think it’s threat to everybody’s personal life*.” (P15, 24, Male)

Another perspective revolved around data integration. One participant suggested that linking the ABDM with existing national identifiers like Aadhaar could streamline processes and minimize the burden of managing multiple cards. However, they also acknowledged that the adoption of the ABDM would require time, given the saturation of existing identification systems and the need for trust in its implementation.

The participants highlighted a delicate balance between the utility of digitized health records and the challenges posed by data security and privacy concerns. Although the potential benefits of the ABDM, particularly in record management and accessibility, were widely acknowledged, privacy concerns—particularly regarding the handling of sensitive information—were identified as a significant barrier to its acceptance and implementation. Addressing these concerns with strong data protection frameworks, clear communication about privacy safeguards, and optional data-sharing mechanisms will be critical for the broader acceptance of the ABDM.

### 7.5. Theme 5: User Adoption and Usage of the App

The responses of the study participants revealed a multifaceted interplay among the factors influencing their adoption decisions, including trust, perceived necessity, and concerns regarding data security. These findings highlight the importance of addressing user concerns and enhancing trust in the ABDM app to encourage broader adoption among the target population. Efforts to improve the awareness of the app’s benefits and functionalities may help to overcome barriers to adoption among individuals with existing healthcare coverage and those with limited perceived needs for health tracking.

The participants expressed familiarity with the ABDM, having explored its features on the website and found the materials informative. However, despite recognizing its value, a few participants hesitated to download the app due to already being covered by a state-run insurance scheme and concerns about legal policies.

“*I have trust issues like. I did not. I downloaded it OK, but then they asked Aadhar number, and I uninstalled it*.” (P17, 32, Female)

A recurring concern was data security and trust, particularly regarding the requirement to link the app with Aadhaar. Some participants expressed reluctance to proceed with downloading or using the app due to apprehensions about sharing sensitive personal data. One participant explicitly noted uninstalling the app upon encountering the Aadhaar linking requirement, citing a lack of complete trust in the platform. This reflects broader concerns about privacy and the perceived safety of digital systems, which can act as significant barriers to user adoption.

“*Yeah, but if it is needed means I’ll download. Otherwise, I don’t go for downloading every app until unless it is necessary., I’m having doubt issue of that whether it is safe or not*.” (P5, 29, Female)

Other participants had similar experiences, indicating a selective approach to app downloads based on necessity and concerns about data security. Another barrier identified was the lack of an immediate need for the app among certain users. Some participants, particularly those who did not frequently seek healthcare services, did not perceive the app as essential in their current circumstances. This suggests that user adoption is likely to be influenced by the relevance of the app to an individual’s specific healthcare needs at a given time. A participant acknowledged that they might consider using the app in the future when healthcare tracking becomes more relevant to them. They expressed a willingness to download the app if deemed necessary but highlighted apprehensions about the safety of downloading numerous apps from the app store.

### 7.6. Theme 6: Drawbacks and Concerns

A participant pointed out that, while they are aware of the facilities offered under the ABDM, they could not access them due to eligibility restrictions tied to their state’s health initiatives. They acknowledged the existence of the ABDM as a parallel initiative by the central government. However, they highlighted the exclusivity of their entitlement to the state-specific healthcare card, indicating a lack of eligibility for the ABDM. Despite their awareness of the ABDM’s facilities, they emphasized the unavailability of this option in their region, highlighting the distinct nature of state and central government healthcare programs. Different states have their own healthcare initiatives and programs, leading to a lack of uniformity and potentially unequal access to healthcare services across the country. Some of the participants highlighted inaccessibility to central schemes like the AB-PMJAY due to eligibility restrictions from overlapping state-run health initiatives. This overlap and lack of integration between state and central schemes creates barriers to equitable access and reduces the perceived inclusivity of the ABDM platform for residents of such states.

The participants expressed concerns about the digitization of health records, highlighting potential threats to personal privacy. They emphasized that certain sensitive medical information should remain confidential and under individual control. They emphasized the risks of revealing such sensitive information online, even with assurances of data security, as no system can guarantee complete protection against breaches.

“*Everyone knows about Ayushman Bharat Digital Mission right? But no one knows why I have to use the app because it doesn’t provide me anything*.” (P12, 25, Male)

The overall data suggest a twofold challenge: while there is limited awareness of the Ayushman Bharat initiative, there is also a lack of understanding or perceived benefit regarding the associated app. This indicates a broader gap in knowledge and engagement with the initiative. Additionally, the lack of understanding or perceived benefit regarding the app highlights the need for clear communication about its purpose, its features, and how it can benefit users.

## 8. Discussion

The participants’ remarks suggest a disconnect between awareness of the initiative and an understanding of its practical implications or benefits for users. Despite their general awareness of the mission, the participants questioned its relevance to their needs, citing a disconnect between the app’s offerings and their healthcare expectations. For individuals who do not directly benefit from the app, or for whom the app does not add significant value compared to existing healthcare services, adoption becomes less appealing. This knowledge gap contributes to low engagement with or utilization of the app, as users fail to see its relevance or value in addressing their healthcare needs. Addressing both the awareness gap regarding the ABDM initiative and the understanding gap regarding the associated app is crucial in fostering greater engagement and utilization among the target population. This requires comprehensive communication and education strategies tailored to the needs and preferences of diverse user groups.

The results also revealed a significant knowledge gap, with many participants confusing the ABDM with the Ayushman Bharat health insurance scheme. This misconception emphasizes the need for more effective communication strategies to clearly differentiate between the two initiatives and emphasize the digitalization of health records, which is the primary goal of the ABDM. Countries like the UK and Australia emphasize digital literacy and healthcare education in school curricula to foster early awareness [30]. Programs that introduce concepts like personal health management and EHRs enable students to act as informed healthcare users. The primary objective within an educational community is to foster deep engagement among students with telemedicine and e-health, cultivating a culture of trust and facilitating the seamless dissemination of vital knowledge [31]. In addition, social media platforms are becoming increasingly integral to modern communication, making them effective tools to reach a broad and diverse audience. As the majority of the population, particularly younger individuals, engages actively with these platforms, they offer an opportunity to increase the visibility and recognition of the ABDM.

Despite a general interest in healthcare advancements and digital tools, the lack of targeted awareness campaigns has resulted in a substantial portion of students remaining uninformed about the mission’s objectives and benefits. Ensuring data privacy is also paramount in healthcare initiatives such as the ABDM; it is crucial to alleviate apprehensions regarding the online exposure of sensitive medical data. The healthcare sector is a prime target for health information theft, and it has historically lagged behind other leading industries in protecting vital data [32]. Incorporating blockchain technology in healthcare could significantly enhance the efficiency and security, eventually benefitting both patients and providers [33,34]. Recognizing the constant evolution of digital healthcare is vital, as advances in technology provide new security and privacy problems. To ensure the privacy and safety of digital healthcare systems, it is critical to stay informed about emerging threats, regulations, and best practices [35]. Considering the pivotal role that healthcare providers play in increasing the awareness and uptake of ABDM services, a strategic approach could involve incentivizing and training these providers to incorporate and promote the ABDM as part of their routine care. However, prior to implementing this strategy, it is essential to qualitatively evaluate healthcare providers’ acceptance and identify the barriers and facilitators that impact the integration of the ABDM’s services into their workflows. Regional disparities in accessing the ABDM highlight the importance of integrating national and state healthcare programs to ensure uniform access and improve overall healthcare delivery and accessibility. It is important to mention that India’s healthcare system is shaped by significant geographical and structural inequalities, such as income levels, urban–rural divides, and inadequate digital infrastructure. The effectiveness of digital health efforts such as the ABDM is impacted by the fact that many rural and border regions have inadequate access to medical services and internet connectivity, whereas metropolitan areas have higher levels of digital adoption and advanced healthcare facilities. This disparity is exacerbated by socioeconomic disparities, which makes affordability, knowledge, and digital literacy key barriers. These discrepancies could be better understood through a quantitative comparison of ABDM adoption by region, which would assist policymakers in ensuring fairer access to digital healthcare across the country.

To efficiently utilize and harness the potential of digital technologies for healthcare advancements, administrators and healthcare workers need to incorporate digital health literacy and advanced digital skills as crucial abilities. These skills enable healthcare professionals to use digital tools effectively, ultimately enhancing patient care [36,37]. Understanding the root causes of their lack of awareness and negative perceptions towards the ABDM is essential in crafting effective interventions to boost its adoption. Tailoring digital processes and solutions to meet user requirements significantly enhances the ABDM. By incorporating end users and key stakeholders into the co-design process, the ABDM can ensure that its solutions effectively address real-world healthcare concerns and preferences. This collaboration will identify possible difficulties early, resulting in more user- friendly and efficient processes. This approach will enhance the relevance and usefulness of the ABDM’s digital health efforts, develop user ownership and satisfaction, and eventually contribute to better engagement with healthcare services.

## 9. Strengths and Limitations of the Study

The main strength of this study lies in its diverse participant sample, which included people between the ages of 23 and 32, with balanced gender representation (10 women and 7 men) and a range of regional origins, including Tamil Nadu, Maharashtra, Kerala, and Karnataka. Moreover, young postgraduate students and early-career professionals were the target audience, ensuring viewpoints from future healthcare professionals and practitioners who are flexible in adopting digital healthcare services. 

Potential biases in the study may stem from the participants’ responses, particularly due to limited digital literacy, which could have influenced the accuracy and completeness of the data collected. Furthermore, the focus on postgraduate students may limit the generalizability of the findings, as it excluded perspectives from other critical stakeholder groups, such as older adults and individuals from rural populations. This narrow scope may result in an incomplete understanding of the broader challenges and opportunities associated with the ABDM.

## 10. Conclusions

The ABDM stands as a pivotal initiative in India’s journey towards a digitally transformed healthcare system. By aiming to create a comprehensive digital health ecosystem, the ABDM seeks to enhance accessibility, improve patient outcomes, and streamline healthcare delivery across the nation. While the mission aligns with global trends in digital health, emphasizing patient empowerment, interoperability, and the integration of technology, it also faces unique challenges, such as limited public awareness and misconceptions about its objectives.

Future healthcare professionals play a role in the successful implementation of digital health initiatives. By fostering a deeper understanding of the ABDM and its functionalities, educational institutions can equip students with the knowledge and skills necessary to advocate for and utilize these technologies effectively in their future practices. To realize the full potential of the ABDM, it is crucial to address these challenges through targeted communication and education strategies that inform the public about the benefits and functionalities of digital health infrastructure. By doing so, the ABDM could play a pivotal role in advancing progress towards universal healthcare coverage and enhancing health outcomes by increasing the efficiency and inclusivity of healthcare services countrywide.

## 11. Recommendations

For future research, it is recommended to conduct quantitative studies aimed at evaluating patient-centric outcomes resulting from the implementation of the ABDM. These studies should focus on assessing improvements in healthcare access, quality of care, and patient satisfaction attributed to the ABDM’s initiatives. Longitudinal assessments could offer valuable insights into the sustained impact of the ABDM on healthcare delivery and patient outcomes over an extended period. Such research endeavors will provide crucial evidence to inform policy decisions and optimize the ABDM’s strategies to achieve long-term healthcare goals in India.

## Figures and Tables

**Table 1:** Participant profile.

Participant ID	Age	Gender	Home Region	Educational Background	Experience in Using ABDM
P1	28	Male	Maharashtra	Bachelor of Physical Therapy	Yes
P2	32	Female	Karnataka	Bachelor of Dental Surgery	Yes
P3	27	Female	Karnataka	Bachelor of Dental Surgery	No
P4	26	Female	Kerala	Cardiac Perfusion Technology	No
P5	29	Female	Karnataka	Bachelor of Dental Surgery	Yes
P6	30	Male	Kerala	English Literature	No
P7	24	Female	Maharashtra	Bachelor’s in Physiotherapy	No
P8	23	Female	Karnataka	Bachelor’s in Hospital Administration	Yes
P9	28	Male	Karnataka	Bachelor of Medicine, Bachelor of Surgery	Yes
P10	26	Female	Karnataka	Bachelor of Dental Surgery	Yes
P11	29	Male	Karnataka	Bachelor of Dental Surgery	Yes
P12	25	Male	Tamil Nadu	Bachelor of Pharmacy	Yes
P13	26	Female	Kerala	Bachelor of Dental Surgery	Yes
P14	27	Female	Tamil Nadu	Bachelor of Homeopathic Medicine and Surgery	Yes
P15	24	Male	Tamil Nadu	Bachelor of Naturopathy and Yogic Sciences	Yes
P16	31	Female	Karnataka	Bachelor of Technology (BTech) in Mechanical Engineering	Yes
P17	23	Female	Kerala	English Literature	No

**Table 2:** Main themes and sub-themes that emerged from the data.

Main Theme	Sub-Themes
ABDM Overview and Awareness	General Awareness Knowledge Gaps Digital Literacy Engagement Levels Implications for Future Healthcare Providers
Advantages and Benefits of ABDM	Paperless Records and Data Accessibility Improved Emergency Response Enhanced Equity and Accessibility Interoperability and Efficiency
Public Awareness and Education Gaps	Use of Social Media for Awareness Campaigns Targeted Awareness through Educational Institutions Integrating Digital Health Education in Academic Curriculum
Data Security and Privacy	Sensitivity of Health Data Willingness Based on Perceived Utility
User Adoption and Usage	Trust in Data Security Perceived Utility and Relevance Conditional Adoption
Drawbacks and Concerns	Exclusions Perceived Lack of Relevance Communication Gap in Benefits

## Data Availability

The raw data supporting the conclusions of this article will be made available by the authors on request.

## Supplementary Materials

The following supporting information can be downloaded at: https://www.mdpi.com/article/10.3390/healthcare13040382/s1, Awareness of and challenges in utilizing the Ayushman Bharat Digital Mission for healthcare delivery: Qualitative insights from university students in coastal Karnataka in India.
