# Supplementary material for: Awareness of and Challenges in Utilizing the Ayushman Bharat Digital Mission for Healthcare Delivery: Qualitative Insights from University Students in Coastal Karnataka in India
Source: Healthcare (Basel). 2025 Feb 11;13(4):382. doi: 10.3390/healthcare13040382 (PMC11855927; doi:10.3390/healthcare13040382)
Supplement: Supplementary file 1 [file healthcare-13-00382-s001.zip › healthcare-3409051-supplementary.pdf]

Awareness of and challenges in utilizing the Ayushman Bharat Digital Mission for healthcare delivery: Qualitative insights from university students in coastal Karnataka in India

Introduction:

Hello, my name is (second author) and I am a researcher at (institution name) conducting a study on the "Awareness of and challenges in utilizing the Ayushman Bharat Digital Mission for healthcare delivery: Qualitative insights from university students in coastal Karnataka in India". This study aims to explore participants' knowledge, experiences, and perspectives regarding ABDM, with a focus on identifying potential barriers to adoption and areas for improvement. Your insights will be invaluable in understanding how young professionals engage with digital healthcare initiatives and what can be done to enhance their accessibility and effectiveness.

This interview is expected to last around 40-50 minutes, and your participation is entirely voluntary. There are no right or wrong answers, and you are free to skip any question or withdraw from the interview at any point. To ensure accuracy in data collection, I would like to seek your **consent to record this conversation**. The recording will be used solely for research purposes, and all responses will remain **confidential and anonymous**. Do I have your permission to proceed with the recording?

Opening Questions:

Could you please tell me something about yourself? (education background, specialized in health/medical science at UG, work experience, family background)

How do you access healthcare facilities (probes: health insurance, monitoring App, Android / iOS User)

Awareness and Familiarity with ABDM

1) How would you like to describe the level of familiarity with the Ayushman Bharat Digital Mission (ABDM) initiative of Government of India? (Probe: first hear about ABDM, received any formal or informal information)

2) Have you or any family member used ABDM app? Do you recall any experiences that you can share with us? (What were the key features, difficulties in navigating the platform)

3) Can you share thoughts on the relationship between Digital Health ID and Aadhaar? Can you explain? (Probes: purpose of linking, concerns in linking)

4) As an end user, what do you think are the benefits of ABDM App? Do you think this will benefit access to healthcare? (Probes: improve access to healthcare, helpful for people in rural and semi-urban areas)

Barriers and Challenges to Adoption

5) What challenges do you think young people face in adopting ABDM? (Probes: technological challenges, lack of awareness)

6) What do you think are the best ways for government to reach out to young generation for widespread use of ABDM App? (Probes: social media, healthcare professionals)

Concerns About Digital Health Data and Security

7) How do you decide whether an online app is safe to use? (Probes; security features in digital platform, government-run digital platforms)

8) Would you be comfortable in sharing personal health information in an online platform of government of India? (Probes: why feel secure, concerns related to data privacy)

Future Prospects for ABDM

9) How do you foresee the success of ABDM in another 2-3 years? Why / why not? (Probe: what could drive success, what could limit its adoption)

10) What are the suggestions to ensure that the whole country benefits from ABDM?

Closing Question:

Anything else you would like to add about your experiences or thoughts on ABDM?
